# A case report of pulmonary tritrichomonosis in a pig

**DOI:** 10.1186/s12917-017-1242-x

**Published:** 2017-11-23

**Authors:** Yuanyuan Shi, Wei Jiang, Zhiyong Ma, Yafeng Qiu

**Affiliations:** Shanghai Veterinary Research Institute, Chinese Academy of Agricultural Sciences, 518 Ziyue Road, Shanghai, 200241 China

**Keywords:** Tritrichomonosis, Pulmonary infections, Bronchoalveolar lavage, Next-generation sequencing

## Abstract

**Background:**

Tritrichomonads like porcine *Tritrichomonas foetus* (previously named *Tritrichomonas suis*)*,* can commensally live in nasal cavity of pigs, but it is rare to cause pulmonary tritrichomonosis.

**Case presentation:**

A 40-day-old piglet was presented for persistent labor breathing and diagnosed with parasite infections in the lung by analysis of bronchoalveolar lavage (BAL) under microscope. By taking advantage of next-generation sequencing approach, we found 9611 homologous tags belonging to 50 annotated genes of tritrichomonads by analysis of mRNA of the bronchoalveolar lavage with the parasite infection. Furthermore, RT-PCR and DNA sequencing analysis confirmed the presence of the tritrichomonad.

**Findings:**

Here, we report a case of pulmonary tritrichomonosis in a pig. By taking advantage of next-generation sequencing approach, we found 9611 homologous tags belonging to 50 annotated genes of tritrichomonads by analysis of mRNA of the bronchoalveolar lavage with the parasite infections. Furthermore, RT-PCR and DNA sequencing analysis confirmed the presence of the tritrichomonad.

**Conclusion:**

Our results demonstrate that *tritrichomonads* like porcine *Tritrichomonas foetus* can cause lung infections of pigs and reveal that next-generation sequencing is potential to identify rare diseases like pulmonary tritrichomonosis in clinical.

**Electronic supplementary material:**

The online version of this article (doi: 10.1186/s12917-017-1242-x) contains supplementary material, which is available to authorized users.

## Background

Tritrichomonosis is caused by tritrichomonads, the flagellated protozoan parasites, which are found from mammal to reptiles worldwide. Mainly, tritrichomonosis is found in cattle characterized with urogenital diseases, such as abortion, infertility and so on [1]. *Tritrichomonas foetus* (*T. foetus*) is the causative agent of bovine tritrichomonosis, which has been also reported to infect cat and human to lead to feline tritrichomonosis [2] and human tritrichomonosis, respectively. Interestingly, neither feline tritrichomonosis nor human tritrichomonosis is characterized with urogenital problems. For example, in cat, *T. foetus* infections usually lead to diarrhea [3]; in human cases, cholecystitis [4], peritonitis [5], pneumonia [6], and menigoencypholytis [7] have been reported to relate *T. foetus*-like infections. Thus, tritrichomonads are not only a concern for cattle farms but also for public health of animal and human.

Except tritrichomonads from cattle and cats, there are tritrichomonad (previously named *Tritrichomonas suis, T. suis*) from swine, *Tritrichomonas mobilensis* (*T. mobilensis*) from nonhuman primates and *Tritrichomonas augusta* (*T. augusta*) from reptiles belonging to tritrichomonads. Although *T. foetus* and *T. suis* are initially called the different strains of *Tritrichomonas*, to date, according to morphology, cross-transmission experiments, and DNA assays, they should be considered as the different isolates of the same strain [8]. Thus, *T. suis* has been currently named as porcine *T. foetus* [9].

Porcine *T. foetus* can commensally live in nasal cavity and gastrointestinal (GI) tract of pigs. According to early study by Hibler et al. [10], there were 55 of 100 pigs with tritrichomonad infections in the nasal cavity, 41 of 512 pigs with tritrichomonad infections in stomach, 215 of 496 pigs with tritrichomonad infections in cecum. Furthermore, most recently a surveillance in China has shown that infection rates of porcine *T. foetus* in GI tract of pigs are more than 12% [11]. Although there are the considerable infection rates of porcine *T. foetus* in pigs, mostly it doesn’t cause any symptoms.

## Case presentation

**Fig. 1:**
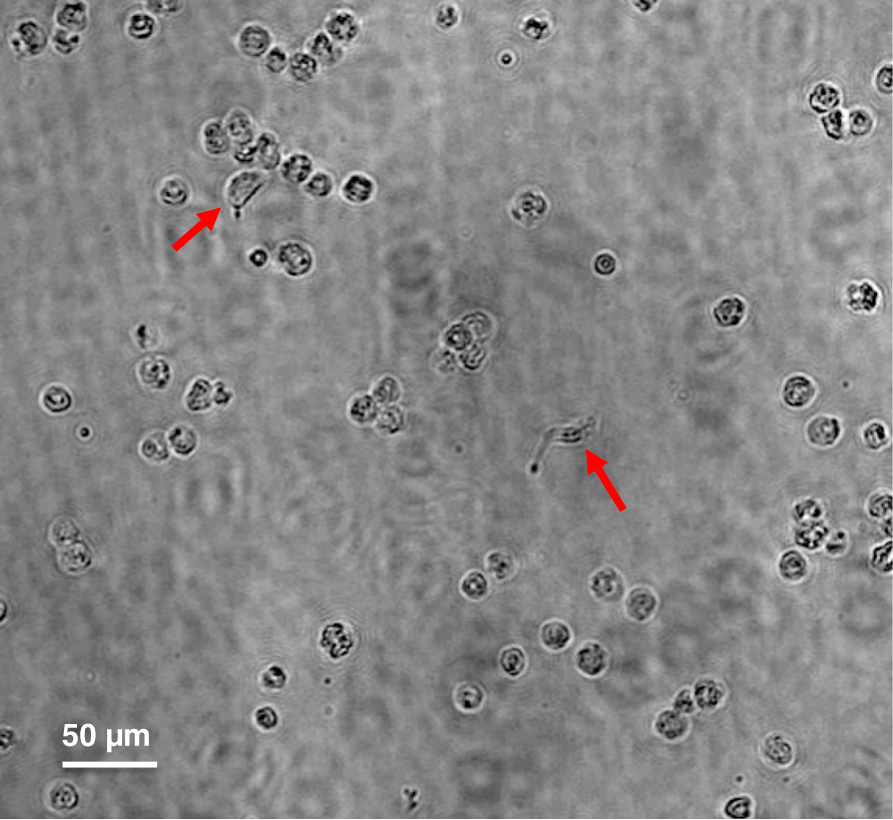
Morphology of leukocytes and the parasite in the BAL sample of a domestic pig under light microscope. Cells were harvested by bronchoalveolar lavage (BAL) from the pig lung, and immediately deposited onto glass slide. Photomicrographs were captured at 20 magnification under light microscope. To note, except the granulocyte, there were the active parasites with flagella (arrow) under microscope

A 40-day-old piglet from a pig farm in Shanghai obviously had respiratory symptoms characterized with breathlessness and diaphragmatic breathing. After necropsy, gross pathology is notable in the lung (not shown). Furthermore, the lung was lavaged and then cells were isolated from bronchoalveolar lavage (BAL) fluid and counted under microscope. During cell counting, surprisingly, we found some living organisms under microscope, which appeared microscler or fusiform, and showed some flagella on both sided of their bodies (Fig. 1; Additional file 1: Video S1), consistent with characteristics of zoomastigophorea.

In order to identify this species, we systemically investigated the BAL sample for digital gene expression profiling (DGE) by next-generation sequencing (NGS) technology, which was based on the Illumina HiSeq™ 2500 platform with single-end sequencing by Major biology (Shanghai, China). A total of 14,732,230 raw tags were obtained after analysis of DGE and 13,550,123 clean tags remained after filtration of erroneous tags. All of the clean tags have been deposited to the NCBI Sequence Read Archive database (SRA) under the accession numbers SRP063389.

Furthermore, to identify specific sequences of the organism, first we removed the sequences (9,190,371 clean tags) belonging to *Sus scrofa* among the clean tags. Totally, we got 4,359,752 unmapped clean tags after removal of sequences of pig. Next, we tested the unmapped clean tags by alignment with nucleotide sequences in NCBI database. As expected, we found some homologous sequences. To note, sequences from *Tritrichomonas* including *T. foetus* and its porcine isolates had been detected by the homology-based analysis. So, based on the preliminary data of sequence alignment and morphological characteristics of the organism, we suspected this organism should belong to *Tritrichomonas.* Though, there are several *T. foetus* transcriptome data available on line [9, 12], unfortunately we cannot do comparative analysis regarding on the difference in sequencing methods among them. Furthermore, the whole genome sequences of *Tritrichomonas* haven’t been released. Subsequently we narrowed our sequence alignment down to nucleotide sequences of *Tritrichomonas* downloaded from the NCBI website (www.ncbi.nlm.nih.gov/nuccore/?term=Tritrichomonas). With only one nucleotide mismatch being allowed, we found 9611 tags were homologous to those of tritrichomonads such as *T. foetus,* porcine *T. foetus*, *T. augusta*, and *T. mobilensis*. After combination of tagged gene information and calculation of copy number of each gene, totally, we got 50 annotated genes of tritrichomonads with different copy numbers (Additional files 2 and 3: Tables S1 and S2). Totally there are 45 genes homologous to those of *T. foetus,* 12 genes homologous to those of porcine *T. foetus,* 2 genes homologous to those of *T. augusta,* and 3 genes homologous to those of *T. mobilensis.* Furthermore*, a*mong those genes, 18S–ITS1–5.8S–ITS2-28S rRNA gene had the greatest copy number, indicating that this gene is highly expressed. Besides, the tags of 18S–ITS1–5.8S–ITS2-28S rRNA gene hit all of *Tritrichomonas* spp*.*, revealing that it is highly conserved in *Tritrichomonas*. Furthermore, our data also showed that Elongation factor 1 alpha (EF-1alpha) and Cytosolic malate dehydrogenase 1 (MDH1) were maybe conserved among *Tritrichomonas* spp. Thus, our RNA-seq data reveal that this parasite belongs to Tritrichomonas.

**Fig. 2:**
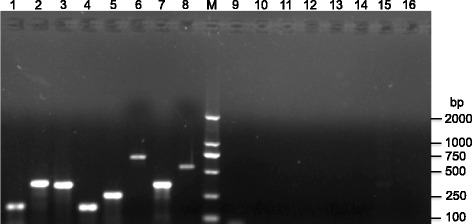
Analysis of PCR amplification products by 2% agarose gel electrophoresis. Genomic DNAs were extracted from BAL sample with Tritrichomonas spp. Infection and control without parasite infection. Lane 1 to 8 were representative of amplification results of gene Enolase, GAP1, CP8, Actin, β-tubulin, CP2, rRNA, EF-1 alpha from infected sample, respectively; Lane 9–16 were were representative of amplification results of gene Enolase, GAP1, CP8, Actin, β-tubulin, CP2, rRNA, EF-1 alpha from control sample, respectively; Lane M, molecular size standard

Furthermore, in order to verify our RNA-sequencing results, we analyzed the BAL sample with the tritrichomonad infections and samples from healthy pig control by RT-PCR. Regarding of gene expression profiles and characteristics of gene conservation, we selected Enolase, GAP1, CP8, Actin, β-tubulin, CP2, rRNA, and EF-1 alpha genes as targets for amplification (Additional file 4: Table S3). Our results showed that positive amplifications were only observed in the tritrichomonad-infected sample, compared with control groups (Fig. 2). Furthermore, we cloned 18S–ITS1–5.8S–ITS2-28S rRNA gene, EF-1 alpha, and CP2 gene to acquire sequence information (data not shown). According to alignment analysis, those genes were close to *T. foetus* and porcine *T. foetus*, consistent with our RNA-seq data. Collectively, these results reveal that the parasite should be considered as *T. foetus* porcine isolate.

## Discussion

Here we reported a pulmonary tritrichomonosis in a domestic pig. In this study, we use the high-throughput sequencing techniques to identify the tritrichomonad lung infection. According to data analysis, totally we got 13,550,123 clean tags from the BAL sample, including 9,190,371 clean tags belonging to *Sus scrofa* and 4,359,752 unmapped clean tags containing the gene information of the parasite. Furthermore, among those of unmapped clean tags, we found 9611 clean tags marking 50 annotated genes of *Tritrichomonas* by homologue gene analysis. As expected, 18S–ITS1–5.8S–ITS2-28S rRNA gene, with the greatest copy numbers among those annotated genes of *Tritrichomonas*, appears conserved among *Tritrichomonas* spp*.* It makes sense that 18S–ITS1–5.8S–ITS2-28S rRNA gene has been chosen to design a PCR-based assay for identification of tritrichomonads [13]. Furthermore, RT-PCR and DNA sequencing data confirmed the tritrichomonad infection in the lung. Overall, our data identify a case of the tritrichomonad lung infection in a pig.

Except PCR and DNA sequencing data, to note, all of these 50 annotated genes were homologous to *T. foetus* including 45 and 12 genes homologous to those of *T. foetus* and its porcine isolates*,* respectively. In comparison, there are only 3 matched genes for *T. mobelinesis* and 2 matched genes for *T. augusta*. Thus, altogether those data showed that the parasite was *T. foetus* porcine isolate. According to the difference in genotype, there are bovine genotype and feline genotype of *T. foetus*. Yet, at this stage, we don’t know which kinds of genotypes the porcine *T. foetus* isolate belongs to.

In clinical, lung infections by trichomonads like *Trichomonas tenax* are common [14]. In comparison, lung infections of *Tritrichomonas spp.* are really rare. According to records, in human, there is one case of *T. foetus* lung infections in a patient with AIDS [6]; in pigs, in 1964, Zhenmin Shan et al. found a case of *Tritrichomonas spp.* respiratory infections in pigs by a morphologic analysis, which were only reported in Chinese (ref not shown). Yet, we don’t know how the tritrichomonad can cause lung infection in the pig. To note, as a commensal of human mouth, most of *T. tenax* lung infections are associated with aspiration in patients with lung disorders including lung cancer, lung abscess or bronchiectasis [15]. And the human case of pulmonary tritrichomonosis showed that the patient was with *Pneumocystis pneumonia* lung infections in spite of AIDS. In our case, except of the tritrichomonad we also found *Pasteurella multocida* and *Trueperella pyogenes* infections in the lung of this pig (data not shown). Thus, it is not excluded that the tritrichomonad is aspirated into the lung under some bacterial lung infections, supporting the notion that trichomonads could not cause pulmonary disease on their own.

## Conclusions

Collectively, we identify a pulmonary tritrichomonosis in a domestic pig by using next-generation sequencing approach. To our best knowledge, it is the first time to explore the gene expression profiles in tritrichomonad-infected lung by using the high-throughput methods. As the pathogens for animals and humans, there is short of information on not only gene sequences of tritrichomonads, but also immune host response to tritrichomonad infections. Future studies by comparing the data from the tritrichomonad*-*infected BAL sample with those from health pig will gain insights into immune response to tritrichomonad infections, special in the lungs, which will provide some basis of understanding host-pathogen interactions.

## Additional files

Additional file 1: Video S1. Morphology of the leukocytes and parasite in BAL. (AVI 75 kb)

Additional file 2: Table S1. The copy number(s) of each homologous tag of tritrichomonads. With only one nucleatide mismatch being allowed, all of unmapped clean reads (4,359,752) agligned with nucleotides sequences of tritrichomonas from the NCBI website. Copy number(s), sequence and homologous gene name of each tag are included in the dataset. (XLS 619 kb)

Additional file 3: Table S2. The copy numbers of the annotated genes of tritrichomonads. After aglinment, all of the unmapped clean reads totally mapped to 50 genes belonged to Tritrichomonas. And the copy number(s) of each homologous gene in four strains and in each strain was calculated, respectively. To note, * means no sequence in NCBI. (XLS 34 kb)

Additional file 4: Table S3. Description of primer sequences and genes used for PCR amplifications. (DOCX 76 kb)

## Abbreviations

AIDS: Acquired immune deficiency syndrome
cDNA: Complementary DNA
DNA: Deoxyribonucleic acid
EDTA: Ethylene diamine tetraacetic
mRNA: Messenger RNA
NCBI: National center for biotechnology information
PBS: Phosphate solution buffer
PCR: Polymerase chain reaction
RNA: Ribonucleic acid
rRNA: Ribosomal RNA
RT-PCR: Reverse transcription PCR
