# Supplementary material for: A case report of pulmonary tritrichomonosis in a pig
Source: BMC Vet Res. 2017 Nov 23;13:348. doi: 10.1186/s12917-017-1242-x (PMC5701426; doi:10.1186/s12917-017-1242-x)
Supplement: Supplementary file 4 — Description of primer sequences and genes used for PCR amplifications. (DOCX 76 kb) [file 12917_2017_1242_MOESM4_ESM.docx]

Table S3. Description of primer sequences and genes used for PCR amplifications

| **Gene name** | **Amplicon size (bp)** | **Acc. number** | **Primer sequence (5′-3′)** |
| --- | --- | --- | --- |
| 18S-ITS1-5.8S-ITS2-28S rRNA | 348 | U85967.1 | CCTGCCGTTGGATCAGTTTCGTTAA/  CGGGTCTTCCTATATGAGACAGAACC |
| Cysteine protease (CP2) | 709 | JX187040.1 | CGAAAGGTCACGGATACACA/  CCCCATGAGTTTCTCACGAT |
| Cysteine protease (CP8) | 343 | JX648163.1 | CAACGCCGCTAAAGGAAAAT/  AGCATCCATAAGACCACCATTG |
| Elongation factor 1 alpha  (EF-1 alpha) | 156 | AB468118.1 | ATGCCCCAGGACACAGAGACT/  GGGTGAAAGCAAGAAGAGCGT |
| Glyceraldehyde-3-phosphate dehydrogenase (GAP1) | 353 | AF022415.1 | CTACCCAACAGAATGCCAAGT/  CTGATGGAACGGAAAGGACG |
| β-tubulin | 248 | AY277786.1 | TCTTCGGACAATCTGGTGCC/  TGGTGATGGGACGATGGAGT |
| Actin | 162 | AB468092.1 | AAGCCCCAATGAACCCAAAG/  GGGAAACACCATCACCAGCA |
| Enolase | 165 | AY277773.1 | CAACAGACGGAACAGAACTCAG/  GGATGTGGAACTTGGCTGGA |
